# PB.2. Axillary node ultrasound in breast cancer: which threshold for 'diffuse cortical thickening' predicts node metastasis?

**DOI:** 10.1186/bcr3713

**Published:** 2014-11-03

**Authors:** P Hamilton, L Clarke, A Leaver

**Affiliations:** 1 Gateshead Hospitals NHS Trust, Gateshead, UK

## Introduction

There is variation in the literature and between breast units in the diffuse cortical thickening (DCT) threshold used for preoperative lymph node needle sampling. In our centre, needle sampling is performed in patients with DCT of 2 mm and above (LN3 of an LN1 to LN5 ultrasound classification). Results triage patients to appropriate axillary surgery: sentinel node biopsy or axillary clearance. This study investigates our 2 mm needle sampling threshold, to determine whether we can safely increase to 2.3 or 3 mm as used in some centres.

## Methods

The authors performed a retrospective audit reviewing multidisciplinary team meeting records and ultrasound images of all invasive breast cancer patients with LN3 nodes diagnosed and operated upon in 2012 and 2013. Positive predictive value (PPV) was calculated for DCT ranges and a post-test probability of a malignant needle sample, and then a surgical yield of greater than two metastatic nodes.

## Results

A total of 223 female patients had an LN3 result and underwent needle sampling. The PPV for a malignant needle sample for DCT in the ranges 2.0 to 2.29 mm, 2.3 to 2.99 mm and ≥3.0 mm were 4.1% (1/24), 10.8% (12/111) and 17.4% (15/86). The PPV for a surgical yield of greater than two metastatic nodes in the ranges 2.0 to 2.29 mm, 2.3 to 2.99 mm and ≥3.0 mm were 16.6% (4/24), 4.5% (5/111) and 9.3%(8/86).

## Conclusion

The 2 mm threshold leads to more accurate patient triage to appropriate axillary surgery, but requires many patients to undergo needle sampling for a small positive yield. Even the 2 mm DCT threshold does not identify all patients with bulky (>2 nodes) metastatic disease.

